# Development of Porcine Accessory Sex Glands

**DOI:** 10.3390/ani14030462

**Published:** 2024-01-31

**Authors:** Trish Berger, Valerie Guerrero, Rosalina Boeldt, Erin Legacki, Megan Roberts, Alan J. Conley

**Affiliations:** 1 Department of Animal Science, University of California, Davis, CA 95616, USA; valeriedguerrero@gmail.com (V.G.); erin.legacki@usda.gov (E.L.); megan.e.roberts@gmail.com (M.R.); 2 Department of Population Health and Reproduction, University of California, Davis, CA 95616, USA; ajconley@ucdavis.edu

**Keywords:** estrogen receptor, prostate, seminal vesicles, bulbourethral glands, GPER

## Abstract

**Simple Summary:**

The male accessory sex glands contribute fluid, proteins, and energy sources to the ejaculate. Although these contributions may not be essential for reproductive success, these accessory sex glands are thought to have positive impacts on reproductive success. By contrast, the abnormal function of the adult prostate is a human health concern. Recognition that postpuberal organ functions are significantly influenced by the internal environment during perinatal development reinforces the motivation to increase our understanding of the early development of these accessory sex glands, particularly in a species like the pig, in which developmental steps can be delineated. The response to reducing the normal concentrations of sex steroids was the focus of these studies on the seminal vesicles, the prostate, and the bulbourethral glands. Surprisingly, the growth of these organs was not affected by a reduction in testosterone signaling during the early neonatal interval. Reduction of estrogen signaling, however, stimulated the early growth of these accessory sex glands. This response appears to be mediated by the nonclassical estrogen signaling pathway. Increased understanding of accessory sex gland development has the potential to improve their postpuberal function and to reduce postpuberal contributions to adverse human health.

**Abstract:**

Accessory sex glands are recognized as targets of human disease and may have roles in reproductive success in livestock. The current experiments evaluated the influences of endogenous steroids on the development of porcine accessory sex glands, primarily in the neonatal period. When the aromatase inhibitor, letrozole, was used to inhibit the production of endogenous estrogens in the postnatal interval, growth of the seminal vesicles, prostate, and bulbourethral glands was stimulated. The weights of seminal vesicles, prostate, and bulbourethral glands approximately doubled at 6.5 weeks of age when the reduction in endogenous estrogens began at 1 week of age (*p* < 0.01). However, by 20 and 40 weeks of age, the weights of accessory sex glands were similar between the letrozole-treated boars and the vehicle-treated littermates indicating the growth stimulation was a transient effect when the treatment interval was short. The presence of both classical nuclear estrogen receptors and the G protein-coupled estrogen receptor in neonatal accessory sex glands indicated multiple signaling pathways might mediate the growth inhibition by endogenous estrogens. The absence of a detectable response when the classical estrogen receptors were blocked with fulvestrant (or when the androgen receptor was blocked with flutamide) suggests that endogenous estrogens act through the G protein-coupled estrogen receptor to inhibit the development of accessory sex glands during this neonatal to early juvenile interval.

## 1. Introduction

Accessory sex glands have long been recognized as steroid-responsive tissues that can become neoplastic in later life, causing significant mortality in men [1,2]. Secretions from these glands are not essential to fertilization since epididymal sperm are capable of fertilization in vivo following artificial insemination in multiple species [3,4,5,6,7,8,9,10,11,12]. However, the infertility in vivo of some rodent ejaculates following the removal of individual accessory sex glands indicates a functional role for these glands [13,14,15]. The proteins, energy substrates, buffering capacity, and, potentially, individual molecules with specific functions provided by these glands may serve a facilitatory role in the fertilization process. Increased cervical sperm transport [5], increased sperm binding to oviductal cells [16], altered epigenetic reprogramming of subsequent embryos [17], and a role in establishing pregnancy after fertilization have also been proposed, although some experimental disparities exist within our current understanding [18,19,20,21,22]. 

Developmental impacts on postpuberal function (and disease risk) [23] underscore the potential of increased understanding of influences on prepuberal growth and maturation. Studies on rodents have indicated that the responses of accessory sex glands to sex steroids in later life are primed by exposure in the neonatal period [24,25]. The influence of androgens on the development of these accessory sex glands, including initial growth during the juvenile interval in rats and mice, has been recognized for decades [26,27,28]. Although the pharmacological effects of neonatal estrogen on the development of accessory sex glands have also been established [29], the potential role of endogenous levels of estrogens is less certain. Estrogen receptor and aromatase receptor knockout mouse models can alter the development of accessory sex glands [30,31,32,33,34], which has been attributed to changes in androgen concentrations, androgen receptors, or prolactin. Moreover, laboratory rodents have a short prepuberal interval, which interferes with a clear separation of developmental events in the neonatal, juvenile, and peripuberal intervals. Hence, the examination of the development of these glands in a model species such as the pig, which has a prepuberal interval sufficiently long to facilitate the separation of developmental events, is advantageous. Normal development in boars includes a brief neonatal window of exposure to elevated androgens and estrogens, followed by a juvenile interval with low sex steroids between approximately weeks 6 and 11 and increasing estrogens and androgens until week 16 of age, when a few spermatozoa are present in the epididymis [35,36,37,38]. The pig possesses an additional, distinct advantage for studies evaluating responses to estrogen, as reducing endogenous estrogen synthesis does not alter androgen levels or the hypothalamic–pituitary axis, including an absence of effect on prolactin [36,39,40,41] that might otherwise confound the interpretation of results in other species.

Our primary objective in these studies was to evaluate the responses of the porcine accessory sex glands (prostate, seminal vesicles, and bulbourethral glands) to endogenous sex steroid concentrations, particularly during the neonatal interval. The second objective was to compare the responses of these three major accessory sex glands. Similar, simultaneous responses would suggest that circulating steroids primarily drive accessory sex gland development. Differences in the timing of the response of specific glands would indicate that the response to circulating steroids is modulated locally by differences in the characteristics of the tissues themselves, perhaps in local steroid metabolism or levels of receptor expression.

Previously, we observed smaller accessory sex glands in postpuberal boars that had maintained a reduction in endogenous estrogen production past puberty [4]. This finding, coupled with initial observations of larger accessory sex glands in juvenile boars treated to reduced endogenous estrogens neonatally, led to the hypothesis that reducing estrogens accelerates the growth and development of accessory sex glands and eventually leads to accessory sex glands that mature earlier, with a smaller final size.

## 2. Materials and Methods

### 2.1. Animal Experiments

All animal experiments were approved by the UC Davis Institutional Animal Care and Use Committee in accordance with the Guide for the Care and Use of Agricultural Animals in Agricultural Research and Teaching. Each replicate within an experiment consisted of littermate boars, with littermates randomly allocated such that one littermate received each treatment, e.g., if there were three treatments, three littermates would be evaluated in each litter. Most boars were of Sygen ancestry and were derived from breeding stock and semen donated by PIC USA (Hendersonville, TN, USA), minimizing genetic variance, although experiments utilizing boars treated from 1 day of age to 1.3 weeks were crossbred with Yorkshire sires. 

### 2.2. Experimental Designs

The designs for all experiments are summarized in Table 1, including treatments and times of tissue collection. The studies investigated the effects of suppressing estrogen synthesis by inhibiting the aromatase enzyme using oral letrozole treatment during the first 1.3 weeks of neonatal development, over multiple intervals during the first 6 weeks, as well as during the immediate peripuberal period (weeks 11–16). All letrozole treatments were given weekly per os at a dose of 0.1 mg/kg, a dose that reduces circulating estradiol concentrations [35,36,39,40,41]. Some of these trials included littermates that were hemicastrated; since hemicastration had minimal effect on accessory sex gland weights, the hemicastration treatment is not discussed. In addition, the antagonism of estrogen receptors was explored using fulvestrant (Tocris USA, Ellisville, MO, USA) delivered by implanted osmotic pumps (2 mL) during weeks 1–6.5 to better explore the mechanisms of the response to reduced estrogen [42]. A second osmotic pump was implanted at 5 weeks of age to continue delivery of this ESR1 and ESR2 antagonist (and GPER agonist) through tissue collection at 6.5 weeks of age, although the last oral dosing with letrozole or vehicle was at 5 weeks of age, as the response to letrozole is sustained for at least two weeks at this age. The equivalent increase in Sertoli cell numbers in the fulvestrant-treated and letrozole-treated littermates compared with the vehicle-treated littermates indicates the fulvestrant treatment was effective in these animals [39]. The influences of androgen receptor activation were also explored using the antagonist flutamide (10 mg/kg bw) administered by daily gavage [43]. The effectiveness of the flutamide dose at the testicular level was verified by the increase in Sertoli cell numbers. 

During tissue collection, the seminal vesicles, prostate, and bulbourethral glands were collected and weighed. A central longitudinal section of the prostrate, a cross-section of the bulbourethral gland near the center of the gland, and the tip of a lobe of the seminal vesicles were fixed in 4% paraformaldehyde in PBS prior to rinsing, dehydration, and embedding in paraffin. Additional tissue aliquots were flash-frozen.

### 2.3. Experimental Procedures

#### 2.3.1. Stromal, Glandular, and Luminal Area Proportions in Seminal Vesicles and Prostate

Ten random fields of a section of prostate or seminal vesicle from an animal were imaged with an Olympus BH2 microscope, 10× objective, QImaging Micropublisher digital camera, and QCapture Pro software (Version 5.1, QImaging Corporation, Surrey, BC, Canada). Outlines of the secretory tissues and the luminal spaces were traced using the NIH ImageJ program (Version 1.4.0, National Institutes of Health, Bethesda, MD, USA, Wayne Rasband). The total pixels of the photo were calculated, and the total area of the glandular tissue and luminal space were subtracted from the total area to determine the stromal area. The percentages of stromal tissue, glandular tissue, and luminal space were calculated for each image and averaged for the 10 images. Three to five littermate pairs were evaluated at each of the designated ages. The intra-assay coefficients of variation (CV) for 10 fields (3 different samples) were 1.5% for stromal tissue, 6.8% for glandular tissue, and 7.4% for luminal space in the prostate at 1.3 weeks of age. The CVs for seminal vesicle tissues from 1.3-week-old animals were 1.1% for stromal tissue, 5.9% for glandular tissue, and 20.0% for luminal space. 

#### 2.3.2. Immunohistochemistry

Seminal vesicles, bulbourethral glands, and prostates were processed for immunocytochemistry as previously described for the testis [44] and as detailed in Table 2. Briefly, 5 µm thick paraffin sections were dewaxed in Citrisolv (Fisher Scientific, Pittsburgh, PA, USA), rehydrated through a graded ethanol series (100%, 95%, 70%), and washed in running water. Sections were subjected to antigen retrieval (5 min steam treatment at 93.3 °C in a citrate-based antigen retrieval solution, H3300 (Vector Laboratories, Burlingame, CA, USA)) followed by blocking of endogenous peroxidase activity during incubation for 30 min with 0.3% hydrogen peroxide in methanol or 10 min with 3% hydrogen peroxide in methanol. Sections were subsequently labeled with antibodies to steroid receptors and markers of development (SRY-box 9 (SOX9), fibroblast growth factor 10 (FGF10), or smooth muscle actin (ACTA2)). Sections to be labeled with ESR2 antibodies were blocked with 20% normal rabbit serum in Tris-buffered saline containing 50 mg BSA/mL followed by blocking of avidin and biotin sites (Vector Laboratories, Burlingame, CA, USA, SP2001), and all other sections were blocked with diluted goat serum (Vector Laboratories PK6101). During incubation with the primary antibody, the negative control sections on the same slide were incubated with diluted normal serum from the same species as the primary antibody. The GPER-labeled sections and corresponding negative control sections were incubated for 30 min with an alkaline phosphatase polymer reagent. Sections labeled with the ESR2 antibody and the corresponding negative control sections were subsequently incubated with biotinylated rabbit anti-mouse IgG (DAKO Corporation, Carpinteria, CA, USA, #E0464). All other sections were incubated with biotinylated goat anti-rabbit antibody (Vectastain Elite ABC kit, Vector Laboratories, #PK6101) and incubation with biotinylated antibody was followed by incubation with ABC reagent. All samples from an individual tissue type were evaluated for a hormone receptor in a single assay. 

#### 2.3.3. Labeling Analysis

The labeling intensity of immuno-reactive SOX9, FGF10, and ACTA2 proteins was evaluated in digital images. A minimum of 6 fields were randomly chosen for each tissue sample, and images were recorded with a 20× objective using an Olympus BH2 microscope with a QImaging Micropublisher digital camera and QCapture Pro software. Four observers blinded to treatment ranked the labeling intensity of the stroma and the secretory epithelium on a 1–3 number scale (with 3 being the darkest and 1 being the lightest). The labeling intensity by antibodies to the steroid receptors was similarly evaluated by two observers blinded to treatment. Scores averaged across observers were subjected to statistical analysis. 

#### 2.3.4. qPCR

The relative expression of GPER was evaluated in the seminal vesicles and prostates from littermate boars using CTCTTCCTGTCCTGCGTCTA as the forward primer and GAAGCGGATGTTCACCACC as the reverse primer. The appropriate size of the product was confirmed on an agarose gel. The RT-qPCR reaction was performed using an Applied Biosystems 7500 fast Real-Time PCR system with a Fast SYBR Green PCR master mix (Cat no. 4385612, Applied Biosystems, Foster City, CA, USA). Primer concentrations were 200 nM for the reference gene *SPAG7* and 150 nM for *GPER*. Dilution curves prepared from aggregated cDNA indicated calculated efficiencies of 100% for *SPAG7* and 96.7% for *GPER* with appropriate melt curves.

### 2.4. Statistics

The weights of accessory sex glands were subjected to analysis of variance using Proc Mixed within SAS 9.3 (SAS Statistical Programs (SAS Institute, Cary, NC, USA)). The litter was considered a random factor, and treatment with letrozole, fulvestrant, or flutamide was considered a fixed factor; a *p*-value less than 0.05 was considered significant. Linear contrasts were used to evaluate treatment differences when more than two treatments were part of the experimental design. Although body weights did not differ between or among treatments, additional analyses for weights of accessory sex glands were performed to verify that body weight as a covariate did not reduce the statistical significance of treatment effects. Additional characteristics of the accessory sex glands were similarly analyzed with Proc Mixed, e.g., time as a fixed factor and litter as a random factor, again with linear contrasts to evaluate differences among time points if more than two time points were included in the analysis.

## 3. Results

### 3.1. Growth and Development in Response to Reduced Endogenous Estrogens

**Figure 1:**
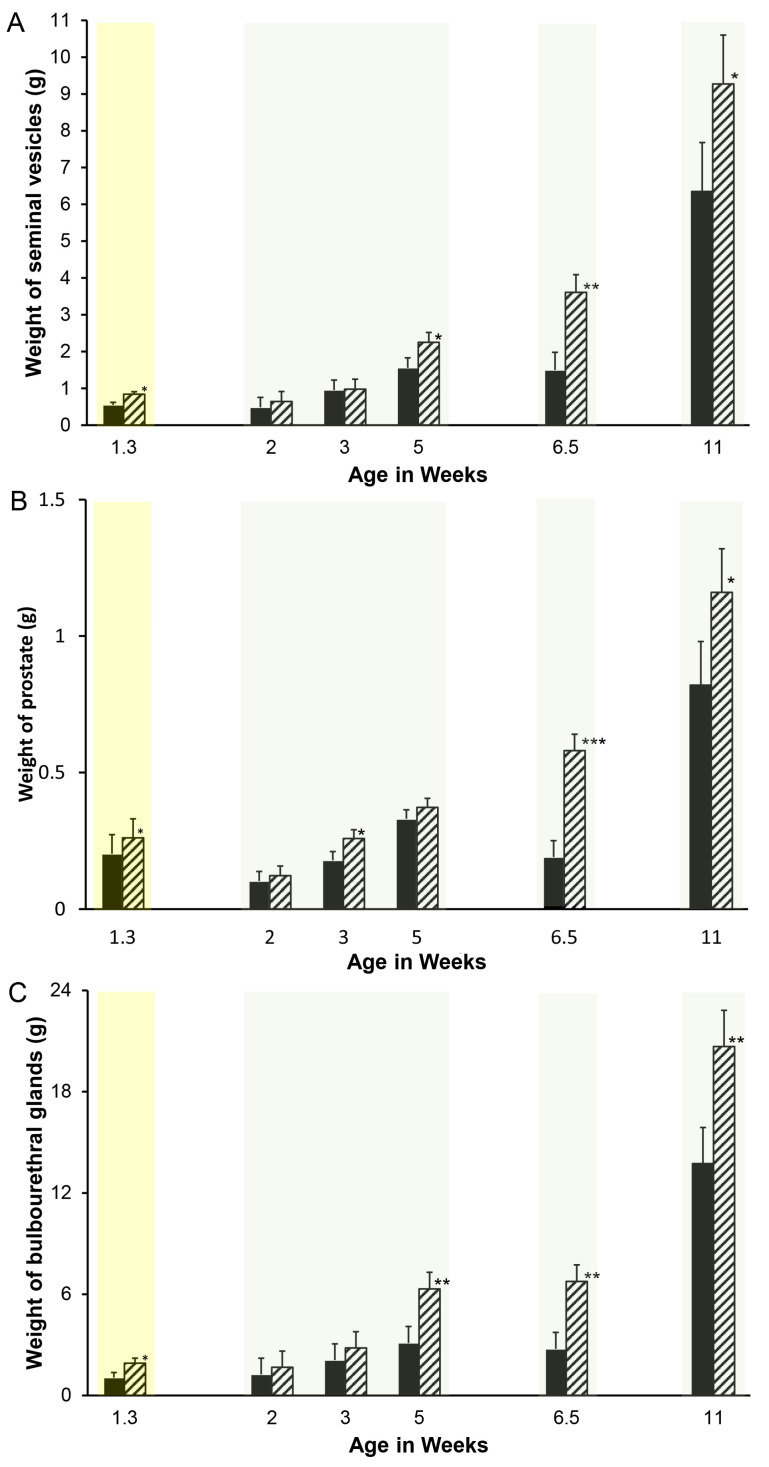
Reducing endogenous estrogens stimulates initial growth of accessory sex glands in pigs. One member of littermate pairs of boars was orally treated weekly with 0.1 mg letrozole/kg bw (hatched bars), and the remaining member was treated with the canola oil vehicle (solid bars). Animals with tissues recovered at 1.3 weeks of age were treated once at 1 day of age; the remaining animals were treated weekly beginning at 1 week of age. Four separate experiments delineated by light background shading consisting of littermate pairs (or littermate sextets for the 2-, 3-, and 5-week experiments) are represented. Values represent least squares means and SEM from the statistical analysis for five animals in each treatment at 1.3, 6.5, and 11 weeks of age and four animals in each age and treatment combination at 2, 3, and 5 weeks of age. * indicates letrozole and vehicle treatments differ with *p* < 0.05; ** indicates letrozole and vehicle treatments differ with *p* < 0.01; *** indicates letrozole and vehicle treatments differ with *p* < 0.0001. (**A**). Seminal vesicles, (**B**). prostate, and (**C**). bulbourethral glands.

**Figure 2:**
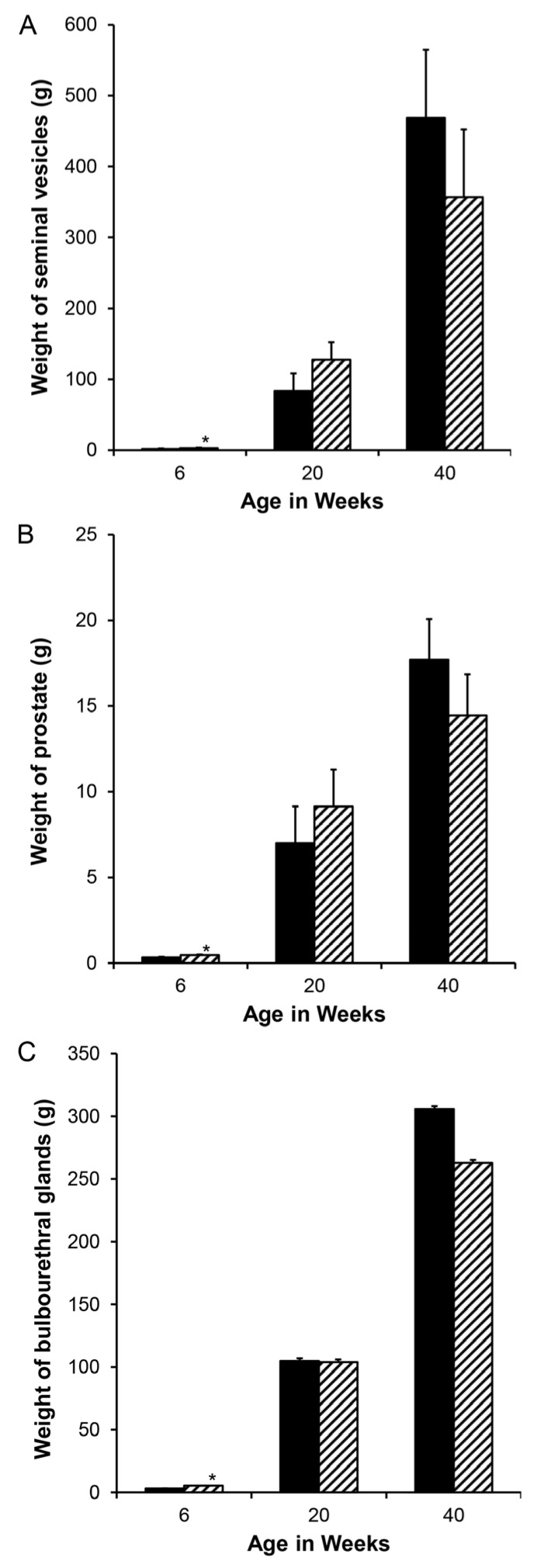
Although reduced endogenous estrogens during the neonatal interval stimulated the initial growth of accessory sex glands and was detectable at 6 weeks of age in these litters (hatched bars represent letrozole treatment), the response did not persist postpuberally when the treatment interval was too short to reprogram aromatase. (**A**). Seminal vesicles, (**B**). prostate, and (**C**). bulbourethral glands. * indicates letrozole and vehicle treatments differ with *p* < 0.05.

Accessory sex gland weights in vehicle-treated boars appeared to increase with age as expected, with the exception of a pause between weeks 5 and 6.5 (Figure 1). Reducing endogenous estrogens beginning at 1 day of age with letrozole increased the growth of the seminal vesicles (Figure 1A), prostate (Figure 1B), and bulbourethral glands (Figure 1C) before 1.3 weeks of age. Reducing endogenous estrogens beginning at 1 week of age increased the weight of the seminal vesicles and bulbourethral glands from 5 to 11 weeks of age (Figure 1A,C) and of the prostate at 3 weeks, 6.5 weeks, and 11 weeks of age (Figure 1B). Boars treated from 1 through 5 weeks of age had increased weights at 6 weeks, as expected from preceding experiments (Figure 2), but littermates did not sustain either the increase in weights of the accessory sex glands through 20 or 40 weeks of age or the reduction in estradiol through 40 weeks. Accessory sex gland weights were not affected in animals treated with the aromatase inhibitor letrozole from 11 to 16 weeks of age during the peripuberal interval (Table 3). 

**Figure 3:**
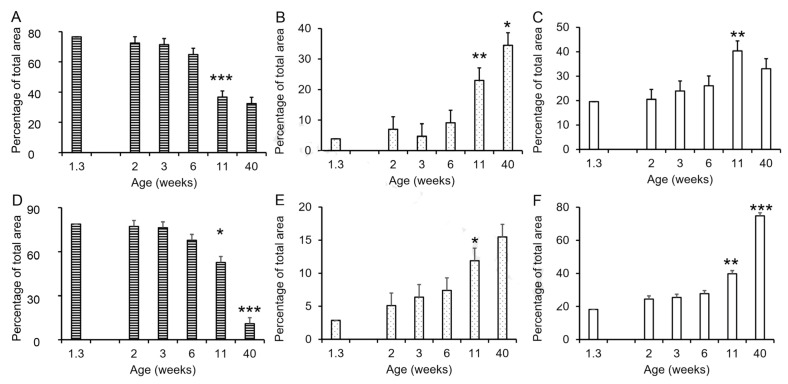
Changes in tissue composition of porcine accessory sex glands during postnatal development. (**A**). Decrease in relative size of stromal tissue compartment during seminal vesicle development. (**B**). Increase in relative size of the luminal compartment during seminal vesicle development. (**C**). Proportion of the seminal vesicle tissue occupied by secretory tissue. (**D**). Decrease in relative size of stromal tissue compartment during prostate development. (**E**). Increase in relative size of the luminal compartment during prostate development. (**F**). Increase in proportion of gland occupied by secretory tissue during prostate development. Values represent the mean of five vehicle-treated boars at 1.3 weeks of age or the least squares mean ± SEM of 3 to 6 vehicle-treated boars with PIC genetic background at older ages. * indicates value is different from preceding time point, *p* < 0.05; ** indicates value is different from preceding time point, *p* < 0.01; *** indicates value is different from preceding time point, *p* < 0.001.

The development of the seminal vesicles and prostate involved changes in the proportions of the stromal, luminal, and glandular compartments (Figure 3). The proportion of stromal tissue decreased significantly between weeks 6 and 11 in both the seminal vesicles and prostate (Figure 3A,D) with minimal change before 6 weeks of age, and the mature proportion was reached at approximately 11 weeks of age in the seminal vesicles and later for the prostate. The proportion of luminal tissue increased between 6 and 11 weeks of age in both the seminal vesicles and the prostate, with the mature proportion approximated by 11 weeks in the prostate and somewhat later in the seminal vesicles. The proportion of glandular tissue also increased between 6 and 11 weeks (*p* < 0.01), although this increase was numerically small in the seminal vesicles. The mature proportion of glandular tissue was reached in the seminal vesicles by 11 weeks of age (Figure 3C); however, corresponding to the continued decrease in stromal tissue in the prostate between 11 and 40 weeks of age, the proportion of glandular tissue in the prostate almost doubled between 11 and 40 weeks of age (Figure 3F). The postpuberal boar prostate is primarily glandular tissue with much smaller proportions of stroma and luminal space (Figure 3). By contrast, the seminal vesicles from postpuberal animals had approximately the same proportions of glandular tissue, stroma, and luminal space. 

The proportional area of glandular tissue increased in the seminal vesicles from letrozole-treated animals at 1.3 weeks of age compared with vehicle-treated littermates (23.3% vs. 19.6%, SEM = 1.1, *p* < 0.05), although proportional areas of the stroma and luminal compartments of these seminal vesicles were not significantly altered. Proportional areas of the glandular and luminal tissue components were also not affected by letrozole in the prostate at 1.3 weeks of age, although letrozole decreased the stromal compartment slightly (63.9% vs. 76.4% in the vehicle-treated animals; SEM = 2.4, *p* < 0.05).

**Figure 4:**
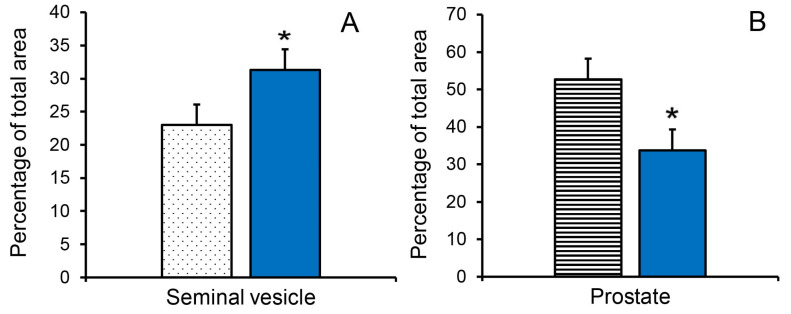
Reducing endogenous estrogens stimulated transitions to more mature tissue composition of accessory sex glands at 11 weeks of age. (**A**). Proportion of luminal tissue in the seminal vesicles was increased in letrozole-treated littermates (blue bar). (**B**). Proportion of stromal tissue in the prostate was reduced in letrozole-treated littermates (blue bar). Values are means ± SEM of five littermates. * indicates letrozole and vehicle treatments differ with *p* < 0.05.

The increased seminal vesicle weight in letrozole-treated boars at 11 weeks of age was accompanied by an increase in the proportion of luminal area (Figure 4A). The letrozole-induced increase in prostate weight was accompanied by an increase in the proportion of glandular tissue at 11 weeks of age (55.1% vs. 37.2%; SEM = 4.0, *p* < 0.05) and a decrease in the proportion of stromal tissue at 11 weeks of age (Figure 4B). 

### 3.2. Immunohistochemical Labeling

**Figure 5:**
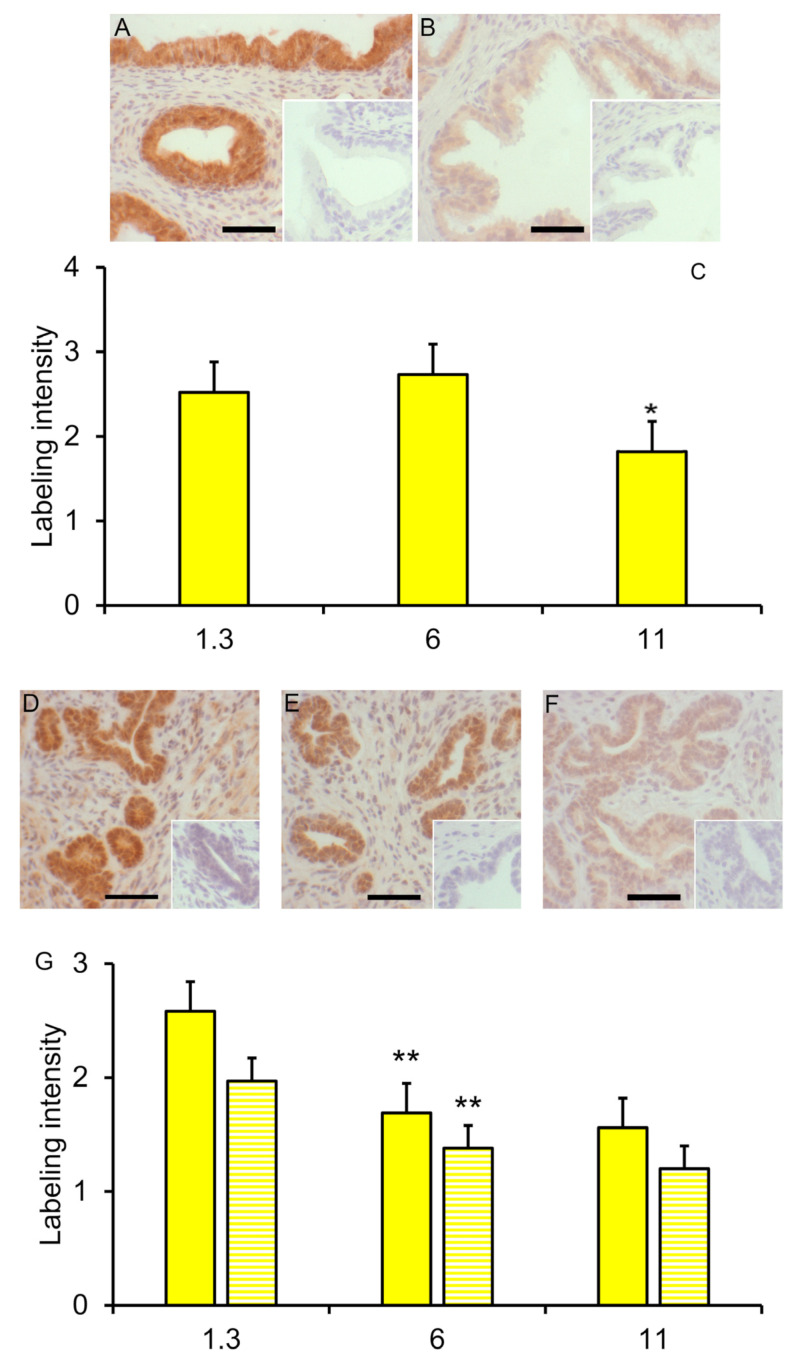
Reduced SOX9 labeling with maturity in the seminal vesicles and prostate. SOX9 labeling in seminal vesicles from 6-week boars (**A**) and 11-week boars (**B**). Insets represent tissues incubated with normal serum rather than SOX9 primary antibody. (**C**). Values represent least squares mean intensity and SEM for glandular epithelium of seminal vesicles for 3 to 6 boars. * indicates a value less than that at the preceding time point, *p* < 0.05. (**D**). SOX9 labeling in both the glandular epithelium and the stroma was clearly visible in the prostate from 1.3-week-old boars. (**E**). Sox9 labeling was less prominent in 1.3-week-old littermates treated with letrozole. (**F**). Sox9 labeling intensity was less prominent but visible in prostates from 6-week-old boars. Insets represent tissues incubated with normal serum rather than SOX9 antibody. (**G**). Solid bars represent least squares mean intensity of glandular tissue, and striped bars represent least squares means of stroma tissue of prostate from 3 to 6 boars. ** indicates a value less than that at the preceding time point, *p* < 0.01. Bars represent 50 microns.

SOX9 immuno-reactivity decreased (reduced labeling intensity by SOX9 antibody) in the glandular epithelium of the seminal vesicles between 6 and 11 weeks (*p* < 0.05) but not earlier (Figure 5A–C); the labeling intensity in the stromal compartment of these seminal vesicles from vehicle-treated boars was low and tended to decrease slightly from 1.3 weeks of age to 11 weeks of age (*p* < 0.10). No effect of letrozole treatment on labeling intensity in the seminal vesicles was detected. SOX9 immunoreactivity in the prostate glandular epithelium and stroma decreased between 1.3 and 6 weeks of age (*p* ≤ 0.01; Figure 5D,F,G) with minimal further change at 11 weeks of age. The letrozole-treated littermates had less immuno-reactivity (lower intensity of labeling) in the stroma at 1.3 weeks of age (*p* < 0.05; Figure 5D,E,G). 

Labeling by the FGF-10 antibody was relatively low in both the prostate and seminal vesicles from the initial observations at 1.3 and 2 weeks of age through 40 weeks of age, without a clear age-related change. Although the letrozole-treated animals had more intense FGF10 labeling of the glandular epithelium of the seminal vesicles at 6 weeks of age compared with their vehicle-treated littermates (labeling intensity score of 1.8 vs. 1.1, *p* < 0.05), no other treatment differences were observed. 

The presence of smooth muscle actin was observed in the stroma of both the prostate and seminal vesicles between 3 and 40 weeks of age, but no age-related changes in the intensity of labeling were suggested, and letrozole-treated animals and their vehicle-treated littermates had similar labeling.

**Figure 6:**
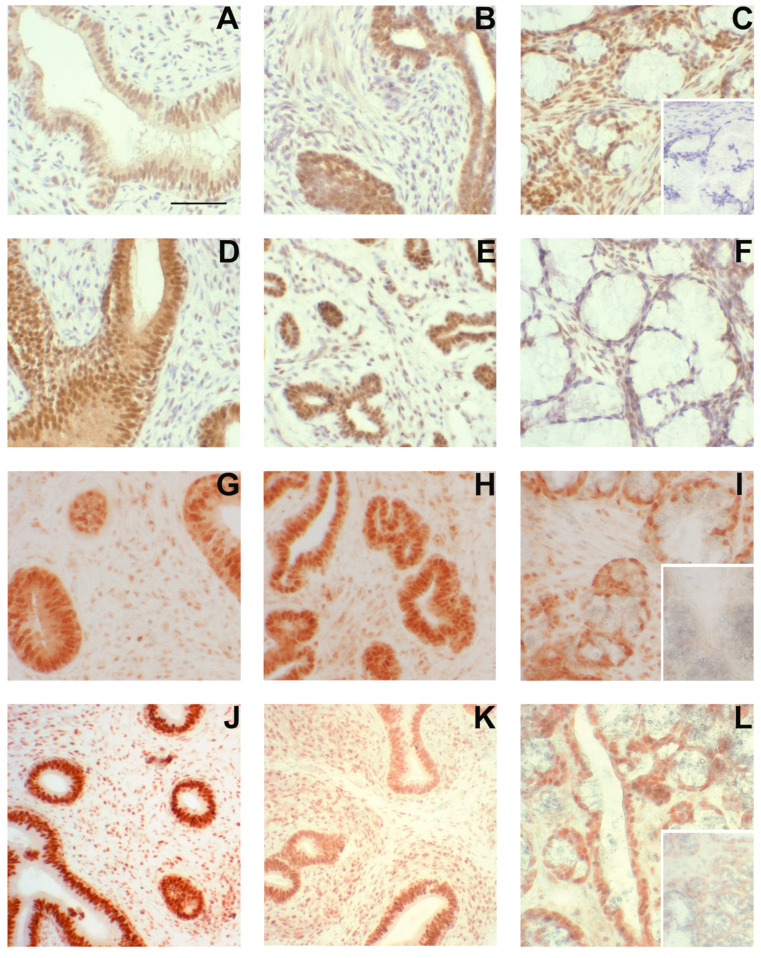
Steroid receptor presence in accessory sex glands from 1.3-week-old boars. Estrogen receptor β (ESR2) immunolabeling (**A**–**F**), estrogen receptor α (ESR1) immunolabeling (**G**–**I**), and androgen receptor (AR) immunolabeling (**J**–**L**) in accessory sex glands from 1.3-week-old boars. Representative sections of seminal vesicle gland from vehicle control boars (**A**,**G**,**J**) and letrozole-treated boars (**D**) indicate that all three steroid receptors are present and letrozole treatment increased immunolabeling intensity of ESR2 in the glandular epithelium. Representative sections of prostate glands from vehicle control boars (**B**,**H**,**K**) and letrozole-treated boars (**E**) indicate that all three steroid receptors are present. Representative sections of bulbourethral gland from vehicle control boars (**C**,**I**,**L**) and letrozole-treated boars (**F**) indicate that all three steroid receptors are present and letrozole-treatment reduced immunolabeling intensity of ESR2 in the bulbourethral gland epithelium. Bar represents 50 µm for all images.

**Figure 7:**
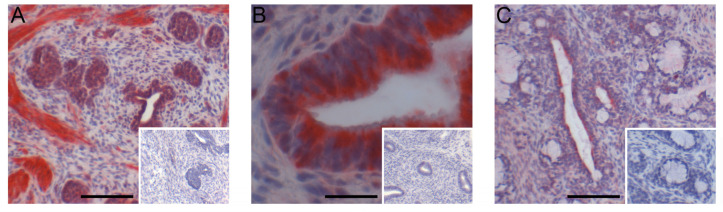
Immunohistochemical localization of GPER in seminal vesicles (**A**), prostate (**B**), and bulbourethral glands (**C**) from 1.3-week-old vehicle-treated boars. Bars represent 25 µm in the seminal vesicles (**A**) and 100 µm in the prostate (**B**) and bulbourethral glands (**C**).

ESR1 was readily detectable in the nuclei of cells from the seminal vesicles and bulbourethral glands at 1.3 weeks of age (Figure 6). The prostate exhibited nuclear labeling as well as cytoplasmic labeling of epithelial cells. ESR2 labeling of cell nuclei was readily detected in the bulbourethral glands and prostate glands from vehicle-treated boars and only faintly present in the nuclei of the seminal vesicle epithelium from the vehicle-treated boars. By contrast, ESR2 labeling was very faint in the bulbourethral gland epithelium of the letrozole-treated boars and intense (including some cytoplasmic labeling) in the seminal vesicle epithelium of letrozole-treated boars. The ESR2 labeling in the prostatic tissues from letrozole-treated boars was similar to that observed in the vehicle controls, and the labeling of ESR1 was similar in letrozole-treated boars to their vehicle littermates for all tissues. The cytoplasmic G-protein coupled estrogen receptor GPER was present in all three of the accessory sex glands (Figure 7). The most intense labeling by the GPER antibody in the prostate was the smooth muscle, followed by the glandular epithelium; the glandular epithelium was the most intensely labeled compartment of the seminal vesicles. Some of the cells in the epithelium lining the ducts were intensely labeled in the bulbourethral glands at 1.3 weeks of age, and the labeling of the glandular epithelium was minimal. The labeling of the stroma was fairly light in all three glands at this age. 

### 3.3. Response to Estrogen Receptor and Androgen Receptor Blockade

**Figure 8:**
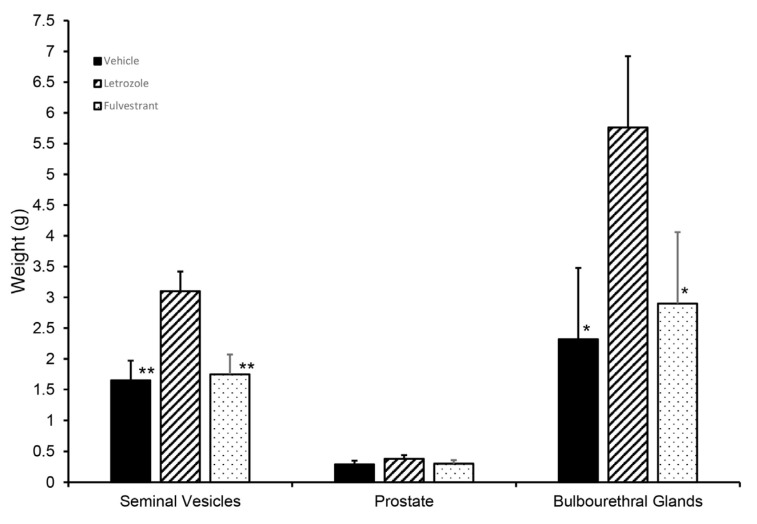
Initial growth of accessory sex glands was stimulated when endogenous estrogens were reduced but not affected if GPER signaling persisted but ESR1 and ESR2 signaling was blocked (fulvestrant). Accessory sex glands were recovered at 6.5 weeks of age and weighed. Values represent least squares means and SEM from the statistical analysis for five animals in each treatment. * indicates values differed from the letrozole treatments with *p* < 0.05 and ** indicates values differed from the letrozole treatment with *p* < 0.01.

**Figure 9:**
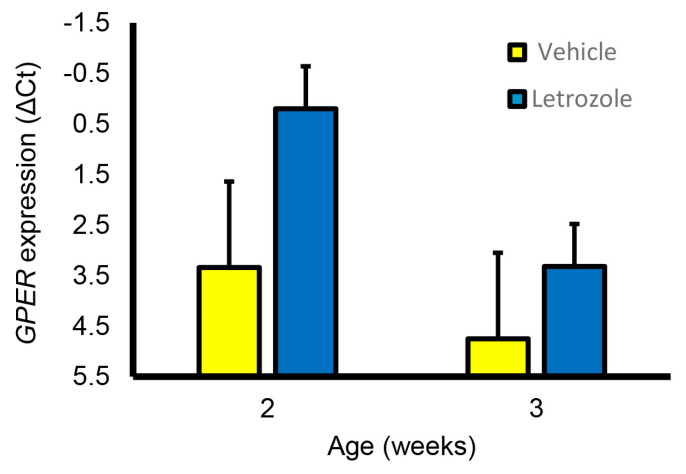
GPER expression was detectable in the prostate at 2 and 3 weeks of age but expression was not affected by letrozole treatment.

Although letrozole treatment increased the seminal vesicle and bulbourethral gland weights at 6.5 weeks, littermates treated with fulvestrant, an ESR1 and ESR2 antagonist, had accessory sex glands similar in size to those from the vehicle-treated littermates (Figure 8). Prostate weights were numerically higher in the letrozole-treated animals compared with the vehicle-treated animals and fulvestrant-treated animals but not statistically different in this experiment. Presence of *GPER* in accessory sex glands was further corroborated by qPCR analysis of prostate and seminal vesicle tissue; however, letrozole treatment did not significantly affect expression in the prostate at 2 or 3 weeks of age (Figure 9).

Seminal vesicle weight, prostate weight, and bulbourethral gland weight were unaltered following the blockade of androgen signaling through the androgen receptor from 1 to 6.5 weeks of age (Table 4). Immunohistochemical labeling revealed that androgen receptors were present in the nuclei of the glandular epithelium and stroma in all these tissues at 1.3 weeks of age, the earliest age examined (Figure 6). 

## 4. Discussion

The results of these studies support four important conclusions. First, endogenous estrogens inhibit the growth of the porcine accessory sex glands between birth and 6.5 weeks of age, coinciding with a time when estrogen concentrations are high or declining [39,41]. Second, the lack of response in the accessory sex glands to ER antagonism by fulvestrant, even though this treatment was equivalent to letrozole treatment in stimulating Sertoli cell proliferation [39], strongly suggests that classical estrogen receptors are not involved in the letrozole-induced increase in accessory sex gland weights. Third, despite the obvious role of androgens in the stimulation of accessory gland growth and function in puberal boars, neonatal glands are not dependent on androgens for development. Since the response to letrozole was absent when treatment was initiated in the peripuberal period, the inhibition of growth by estrogens is developmentally sensitive and restricted to the neonatal period. Lastly, the degree to which glands respond and the timing of their response differ among glands under the same systemic steroid influence, suggesting that responses are modulated locally in some way yet to be determined. This last conclusion is supported by the distinct developmental profiles of mRNA expression in the seminal vesicles and prostate [46]. Developing an understanding of such mechanisms may provide insight into the sensitivity to neoplastic changes in these tissues. 

The continued growth in the seminal vesicles and bulbourethral glands observed as early as 5 weeks of age, and in the prostate at 6.5 weeks of age in letrozole-treated boars, suggests that estrogen secretion by the neonatal testis normally inhibits growth during this period. The reduction in endogenous estrogen without changes in androgens, FSH, LH, prolactin, or inhibin was previously documented for the boars included in this study [35,39,40,41,43]. As previously reported [36], the absence of a hypothalamic or pituitary response to reduced endogenous estrogen is relatively unique to the boar, and insensitivity to estrogens was observed in GNRH-immunized boars as well [47]. Hence, the inhibition results either directly or indirectly from the reduced estrogens or the resulting increase in the androgen-to-estrogen ratio. However, the absence of an effect of flutamide on growth suggests that the increased growth is more likely a response to reduced estrogens alone. Growth inhibition by endogenous estrogens is elicited in the neonatal period and is not evident peripuberally. This observation is consistent with the reported absence of response in accessory sex gland growth or development in boars given estrogen replacement following castration at 10 weeks of age [48]. By contrast, estrogen stimulated the growth of accessory sex glands in the presence of 5alpha-dihydrotestosterone in peripuberal castrated pigs [49] and stimulated accessory sex gland secretion in postpuberally castrated boars [50]. Clearly, accessory sex glands experience developmental windows of sensitivity to both androgens and estrogens, and these windows may be offset in time in individual glands experiencing the same sex steroid exposure. Previously, we reported smaller accessory sex glands postpuberally at 43 weeks of age following letrozole treatment for 12 weeks [4]. In contrast to the treatment through 6 weeks of age, boars in this earlier study had prolonged suppression of aromatase activity and endogenous estrogens, a presumed reprogramming of testicular aromatase, through 43 weeks of age. The reprogramming of testicular aromatase does not occur with boars treated only until 6 weeks of age, which we believe explains the differences between the previous and current results in the eventual postpuberal size of the accessory sex glands. The transient effect of reduced neonatal estrogens on accessory sex gland growth in the boar seems somewhat different from observations in the mouse. Vom Saal and colleagues [51] reported that late fetal exposures had diverse responses on adult prostate weight depending upon the dose of exogenous estrogens. Reduced endogenous estrogen signaling (through ESR1, ESR2, or aromatase knockouts) led to larger adult accessory sex glands in the mouse, although this may be due to changes in other hormones. The absence of effect on other systemic hormones (e.g., FSH, LH, prolactin, testosterone) in all our previous studies in the boar provides persuasive evidence for the inhibition of growth of accessory sex glands by endogenous estrogens during the postnatal interval. 

Either ESR1 and ESR2 might be the signaling pathway mediating the initial growth, as they are both present in at least one compartment of the three accessory sex glands, and interactions between the stroma and the glandular epithelium are recognized as important in regulating growth [52,53]. However, fulvestrant blocks both ESR1 and ESR2 and would mimic treatment with an aromatase inhibitor if this effect was mediated through ESR1 or ESR2. In contrast to the response of Sertoli cells [39], the initial growth of the accessory sex glands was similar in the boars treated with fulvestrant to the vehicle control littermates. This suggested to us that the response might be mediated by GPER. Immunohistochemical localization of GPER to these early accessory sex glands is consistent with GPER mediation of this response, similar to a GPER-mediated proliferation response of the porcine epididymis [45] and GPER presence in the human prostate [54]. The detection of *GPER* by qPCR analysis further supports the presence of GPER in these glands and the hypothesis of GPER involvement. 

We currently hypothesize that endogenous estrogens inhibit initial growth and delay the maturity of these accessory sex glands, while reduced endogenous estrogens stimulate initial growth and accelerate maturity. The presence of secretion in the bulbourethral glands from neonatal boars and in the seminal vesicles from some 11-week-old boars at weights significantly less than mature size suggest that secretion is not an ideal marker for post-development maturity. Changes in the proportions of stromal, secretory, or luminal tissue are another potential marker for accessory sex gland maturity, and age-related reductions in the proportions of stromal tissue were consistent with use as a marker for maturity. These reductions in the proportion of stromal tissue were accompanied by a mild increase in the proportion of glandular epithelium in the seminal vesicles and a more dramatic increase in the glandular epithelium in the prostate with age. The hypothesized acceleration of maturity in the presence of reduced endogenous estrogens is consistent with the observed decreases in the proportion of stromal tissue in the prostate and increases in the proportion of glandular epithelium in the letrozole-treated littermates. Similarly, this hypothesized acceleration of maturity is consistent with the increase in the proportion of luminal space at 11 weeks and in the glandular epithelium at 1.3 weeks in the seminal vesicles from the letrozole-treated littermates compared with their vehicle-treated littermates. Smooth muscle actin was evaluated as a potential marker of maturity [55,56] of these glands, but we detected no difference in labeling intensity with maturity of vehicle-treated animals, so the absence of an effect of letrozole treatment is as expected. 

Fibroblast growth factor 10 decreased with development in murine prostate and seminal vesicles and was no longer readily detected in glands from postpuberal murine males [57]. By contrast, we were not able to detect an age-related change in intensity of FGF10 immunoreactivity in porcine accessory sex glands. Species variation in the response of accessory sex glands is not surprising given the species variation in the relative sizes of individual glands and accessory sex glands as a percentage of body weight [58,59,60]. The transcription factor SOX9 is generally anticipated to decrease with development [61,62], and we observed an age-related decline detectable at 6 weeks in the prostate and at 11 weeks in the seminal vesicles. The initial decrease in SOX9 labeling of the prostate stromal compartment in letrozole-treated littermates is consistent with the hypothesized acceleration of maturity. 

## 5. Conclusions

Increased neonatal and juvenile growth of accessory sex glands was a rapid response to reduced endogenous estrogens in the boar. These experiments also reveal that the increased growth is accompanied by a degree of maturation of the glands. However, the current observations in conjunction with those published previously [4] suggest that a sustained response of the accessory sex glands requires a longer treatment interval, perhaps one long enough to reprogram aromatase activity. 

## Figures and Tables

**Table 1:** Summary of pig ages at treatment and tissue collection ^1^.

Treatment	Animal Age at Treatment	Age at Tissue Collection	Replicates
Letrozole/Vehicle	1 day	1.3 weeks (9 days)	5
Letrozole/Vehicle ^2^	1 week	2 weeks	4
Letrozole/Vehicle ^2^	1–2 weeks	3 weeks	4
Letrozole/Vehicle ^2^	1–4 weeks	5 weeks	4
Letrozole/Vehicle	1–5 weeks	6.5 weeks	5
Letrozole/Vehicle	1–6 weeks	11 weeks	5
Letrozole/Vehicle ^3^	1–5 weeks	6 weeks	5
Letrozole/Vehicle ^3^	1–6 weeks	20 weeks	5
Letrozole/Vehicle ^3^	1–6 weeks	40 weeks	5
Letrozole/Vehicle ^4^	11–15 weeks	16 weeks	5
Letrozole/Vehicle ^4^	11–16 weeks	20 weeks	5
Letrozole/Vehicle ^4^	11–16 weeks	40 weeks	5
Fulvestrant/Letrozole/Vehicle	1–6.5/1–5 weeks	6.5 weeks	5
Flutamide/Vehicle	1–6.5 weeks	6.5 weeks	5

^1^ Littermates were randomly allocated to each treatment in a replicate; hence, the number of replicates is equal to the number of litters. The seminal vesicles, prostate, and bulbourethral glands were collected in each experiment, although there was not necessarily sufficient tissue for immunohistochemistry at the younger ages. ^2,3,4^ Treatments with the same superscript are from the same experiment and harvested at different ages to facilitate age comparisons; these replicates utilized a total of six littermates from each litter.

**Table 2:** List of antibodies used for immunohistochemical labeling.

Antigen	Antibody Source ^1^	Catalog No.	Antibody Dilution	Time, Temperature, and Buffer for Primary Antibody ^2^	Chromagen ^3^	Reference
SOX9	Spring Biosciences	E13470	1:100	Overnight, 4 °C, PBST	NovaRed^®^	
FGF10	Abgent	AP7975b	1:50	1 h, RT, PBS	NovaRed^®^	
ACTA2	Spring Biosciences	E2460	1:150	20 min, RT, PBST	AEC	
ESR2	AbD Serotec	MCA1974S	1:40	Overnight, 4 °C, TBS	DAB	[4,44]
ESR1	Santa Cruz Biotechnology	SC-542	1:250	Overnight, 4 °C, PBS	AEC	[4,44]
AR	Santa Cruz Biotechnology	SC-816	1:1000	Overnight, 4 °C, PBS	AEC	[4,44]
GPER	Aviva Systems Biology	ARP62244	1:100	Overnight, 4 °C, TBS	ImmPACT Red^®^	[45]

^1^ Antibodies were purchased from Spring Biosciences (Pleasanton, CA, USA), Abgent (San Diego, CA, USA), AbD Serotec (Raleigh, NC, USA), Santa Cruz Biotechnology, Inc. (Dallas, TX, USA), and Aviva Systems Biology (San Diego, CA, USA). Immunogen for ESR1 was a mouse peptide sequence and immunogens for all other antibodies corresponded to human peptide sequences. ^2^ Sections were incubated with the indicated dilution of the primary antibody in PBS, PBS containing 0.05% Tween (PBST), Tris-buffered saline (TBS), or TBS containing 20% normal rabbit serum and 50 mg BSA/mL (TBST). ^3^ NovaRed^®^(SK4800), AEC (3-amino-9-ethylcarabazole; SK4200), and ImmPACT Vector Red (SK5105) were all obtained from Vector Laboratories, Burlingame, CA, USA, and DAB (3,3-diaminobenzidine; K3468) was obtained from DAKO Corporation, Carpinteria, CA, USA.

**Table 3:** Reduced endogenous estrogens during the peripuberal interval did not affect accessory sex gland growth.

	Seminal Vesicle wt. (g) ^1^	Prostate wt. (g) ^1^	Bulbourethral Gland wt. (g) ^1^
Letrozole treatment	62.6	4.4	64.2
Vehicle treatment	55.3	4.5	59.1
SEM	16.6	0.7	4.9

^1^ Values represent least squares means for tissues from 5 boars at 16 weeks of age or the SEM from the statistical analysis. Animals were treated from 11 to 15 weeks of age.

**Table 4:** Androgen receptor blockade with daily flutamide treatment during the neonatal interval did not affect accessory sex gland growth.

	Seminal Vesicle wt. (g) ^1^	Prostate wt. (g) ^1^	Bulbourethral Gland wt. (g) ^1^
Flutamide treatment	1.5	0.36	2.0
Vehicle treatment	1.7	0.31	1.9
SEM	0.3	0.04	0.4

^1^ Values represent least squares means for tissues from 5 boars at 6.5 weeks of age or the SEM from the statistical analysis.

## Data Availability

Data are presented within the text of this report.
